# Remote and In-Person Learning: Utility Versus Social Experience

**DOI:** 10.1007/s42979-022-01539-6

**Published:** 2022-12-21

**Authors:** Panos Photopoulos, Christos Tsonos, Ilias Stavrakas, Dimos Triantis

**Affiliations:** 1 Department of Electrical and Electronic Engineering, University of West Attica, Athens, Greece; 2 Department of Physics, University of Thessaly, Lamia, Greece

**Keywords:** Emergency remote teaching, Online, Technology, COVID-19, Synchronous, Asynchronous

## Abstract

The massive transition from in-person to remote teaching increased the impact of technology on the everyday life of the universities. Without the face-to-face component, learning and teaching became a completely different experience for students and teachers. Recording the attitudes and perceptions of the undergraduate students on the new situation became necessary for the faculties to support them effectively. This research collected quantitative and qualitative data from 336 students of all the years of studies. The students preferred in-person teaching and reported higher engagement, learning, and understanding during classroom teaching. More senior students, who had developed face-to-face ties with their colleagues before the pandemic, found it easier to continue their interactions remotely. They were interested in matching learning with the duties and needs at the particular period of their life, despite their beliefs concerning the effectiveness of in-person teaching. The first-year students found it challenging to develop relationships remotely, and they were the most frustrated. Overall, students in the first years of their studies perceived remote teaching as dissatisfactory compared to the more senior students. Similar to other publications, the respondents of this study challenged the effectiveness of remote teaching and the concomitant transition from in-person to remote social relationships.

## Introduction

The period of remote teaching due to the COVID-19 pandemic was a challenge for Higher Education. In March 2020, the Greek ministry of education announced that all the educational activities would go online. The University of West Attica purchased laptops for the academic and administrative staff, organised webinars on educational platforms, and provided a venue for sharing practices, methods and ideas. A few weeks after the COVID-19 outbreak, more than 95% of the undergraduate courses were delivered remotely [1]. The transition from onsite to remote teaching caused a tremendous workload for teachers and students [2, 3]. The teachers had to digitise teaching materials and devise methods to secure learning continuity in the new environment. Before the pandemic, communication with the students was happening all the time. After March 2020, communication became more complicated, requiring consecutive mails to resolve simple issues. The fast transition from in-person to remote teaching gave the impression of continuity in Higher Education. The lectures were delivered remotely and synchronously, the students appeared to follow the lectures, and at the end of the semester, they sat distance examinations. A new order prevailed but with less emotional energy.

Crises and extreme events are not simply overgrown routine events; they require a successful interplay between centralisation and local improvisation [4]. Centralised decisions and guidelines diffuse the expertise of the few knowledgeable individuals across remote sites, allowing local actors to take initiatives on more specific issues. However, during the COVID crisis, the support received from central authorities was somewhat limited [5]. Until the end of the spring semester, June 2020, no COVID-specific quality teaching policy was communicated. The feedback questionnaires administered to the students collected the same information as the years before the COVID-19 crisis. With the Universities closed, traditional feedback mechanisms were barely adequate.

Under these circumstances, a short survey was administered to students of the Department of Electrical and Electronic Engineering to collect information on how they experienced distance teaching. The findings of this survey, presented at the International Conference CSEDU 2021, explored the students’ preference towards face-to-face and remote teaching and studied the variation of the answers with the year of enrolment [6]. This publication revisits the findings of our previous work and expands further on the text answers collected at the same period.

Our research conclusions guided our pedagogical practices during the period of the movement restrictions to support students’ learning endeavour. However, international organisations like OECD and UNICEF promoted the idea of a complete transition of in-person education to online. In some cases, politicians, business leaders, and part of the media further enhanced their vision to articulate persuasive accounts regarding higher education in the post-COVID era [6]. Such descriptions influence the way people understand and interpret reality, and in the case of Higher Education, they affect the interpretation of the research findings. We identify two issues that significantly affect research in education. The first is the researcher’s background beliefs regarding technology, and the second is the relationship between learning and education.

This publication aims to understand students’ modality preferences and the criteria for making preference decisions. Because of the importance of these questions, “Technology and Education” of this publication outlines our understanding of technology. “The Significance of the “No Significant Difference”” discusses the “no significant difference” argument and its importance within a dialogue that overemphasises learning. “Past Research Findings on ERT” gives a snapshot of the literature regarding students’ experiences during the period of emergency remote teaching. “ Methods” comments on the methodology of our research. The results and discussion of the findings are the subjects of “Results and Discussion”, and the publication ends with some conclusive remarks.

## Technology and Education

In Western culture, technology is considered an autonomous entity, which proceeds almost naturally along a predetermined path. From this perspective, technology is an external factor determining our society’s adjustments [7]. Mechanical arrangements, integrated circuits, or computer programs are considered the successful outcome of human efforts to push boundaries back in an endless course of progress [8]. Technology is considered inherently progressive and politically neutral, bringing changes to work and education. It is a force beyond particular interests that dictates changes in society. Society can only take full advantage of technological achievements if education, employment, or healthcare adjust to technology. Technology not only drives but also legitimises change.

Technology has always had an impact on education. Individualised learning has been the aim of technological innovations since 1966 [9]. Computers were introduced in Higher Education in the seventies with the promise to customise education to the individual [10, 11]. Computers were a decentralised technology operating at the faculty or university level to enrich classroom learning [12, 13]. Ideas about replacing the teacher and subsuming the individual learner to the computer system were also circulated in those days. A finely tuned computer system promising improved attention during learning unavoidably led to comparisons between computer-assisted and in-person instruction [11]. Teaching machines, like Autotutor, were used in the UK to “supplement the shortage of specialist mathematics teachers [10], i.e. the replacement of expertise by automation. Regarding the learner, the collection of “information on behaviours such as eye movement or irrelevant body responses during Computer Assisted Instruction” was deemed desirable although unattainable back then [11].

The idea of the supremacy of the automatic systems has been dominant since the beginning of the twentieth century, and it was reheated every time a new technology was considered capable of inflicting the decisive blow to in-person teaching. Around 2000, Information and Communication Technology (ICT) innovations, hand-in-hand with the interest of big corporations to compete in the education market [14–16], revitalised the rhetoric of individualised learning. Courses delivered over the internet [17] became the alternative to classroom teaching. In the new setting, students do not have to commute to the campus or follow lectures in crowded auditoria, but they are responsible for fitting learning into a schedule of personal duties and interests [3]. The teacher’s new role is that of the “Guide on the Side” [18, 19]. References to technology replace the role of the human actors of education in public policy texts [20], educators’ knowledge and skills are subsumed to the technological artefact [21], and the teachers are portrayed as mainly ancillary to students’ learning [16].

Immediately after the pandemic broke out, education companies and platform owners intensified their efforts to expand the EdTech industry and prepare the ground for future profits [14, 22]. During the movement restrictions, they offered their services free of charge to smooth the transition from onsite to any format of non-traditional education. The explosive growth of online education before 2019 [14, 23] was further amplified in 2020 as several EdTech companies treated the pandemic as a business opportunity. The expected increase in capital investments in EdTech [22] was realised and reported in January 2021, showing that during a disruptive year in education, EdTech venture capital investments in the US increased by 30% [24], while globally, this percentage reached 100% [25]. The expansion of existing partnerships between international organisations, prestigious universities, and big corporations [15, 26] allowed private platform owners to penetrate public education further.

Collecting learning data during the transition from onsite to remote teaching was presumably an opportunity for EdTech companies and scholars to record the behaviour of students and teachers during the pandemic [10, 22], although for the latter, collecting information on student experiences during the pandemic was a prerequisite for responding meaningfully to their needs [27, 28].

Technology and automation have a century’s history in education. As Leo Marx has convincingly argued [8], most technologies have the legal status of private property, and individual businessmen, corporate managers, and government officials make vital decisions concerning their design. The corporations invest capital and expertise and anticipate enjoying returns on their investments. Focusing our attention solely on technology usage without considering its design characteristics and ownership status, we remain blind to much that is intellectually and practically crucial.

Based on the above arguments, this publication asserts that low preference for remote teaching does not indicate a problem for technology, nor a lack of understanding or conservativism for the students. Computer-aided instruction does not have to be a substitute for in-person education. Numerous publications have shown that computer-aided instruction can be an effective strategy in learning. They provide examples of technology applications symbiotic with face-to-face education’s social relationships. These are technology applications designed for several purposes, such as: to reduce the administrative burden of large classes, maintain student interest and deliver learning outcomes [29], expand the physical learning space and enrich the learning experience [30], to assist students facing learning difficulties [31] to enrich teaching and learning using simulations [32, 33] and to offer the opportunity to follow the lecture remotely for students who are ill or abroad [34].

Remote teaching can supplement face-to-face education, and it does not necessarily imply a complete transition from education to online learning. For example, virtual microscopes can be used remotely and offer students an engaging learning experience. However, this cannot substitute the experience of using a real microscope during a lab session [32]. Although visiting a virtual Museum is instructive to the students, “The authentic experience of being present at the museum, being able to look around or to be absorbed in a painting, listen to the sounds of the visitors’ talk or whisper and the rattle of coffee cups, the sensation of the hardness of the floor, the temperature in the room, etc., all of this cannot be reproduced through video or image” [30].

## The Significance of the “No Significant Difference”

Jerome Bruner in 1966 described teaching as a call for participation in a process that makes possible the establishment of knowledge. Teaching aims not to produce living libraries but to get students to think. Knowing is a process, not a product. Gert Biesta has resisted the overemphasis on learning, noticing that education is about learning and socialisation and subjectification [35].

Learning is the dominant term in mainstream technology discourse. If online learning is equally effective to in-person learning, it could be an alternative to traditional teaching; otherwise, its benefits would be highly suspected. From this perspective, the possibility of the transition from in-person to online learning requires the firm assurance that there is no significant difference between the two modalities in the learning outcomes. Media comparison studies compare the level of achievement of two groups of learners who have taken the same course delivered by different media. Such research is considered of weak design because student performance is not a question of the medium alone. No medium is inherently better or worse than another; it is the medium and the design of the course which must be considered together [36, 37].

A vital assertion extracted from media comparison studies is the no significant difference phenomenon. According to this, “a large number of media comparison studies have found no statistical difference between learning outcomes of different delivery methods” [17]. Self-selection bias is a problem associated with comparisons between online and in-person learning. In online learning, self-selection bias means that certain factors that influence students’ level of achievement also influence their modality preference. A student’s persistence in learning, engagement with learning, maturity, and human capital endowment, which influence his/her learning achievements, may also influence the choice of a particular modality. Students who choose online courses are probably more comfortable in that format and perform better [17, 36]. Self-selection bias is a point for attention in education and other research fields [38]. There is evidence suggesting that self-selection bias undermines the meaningfulness of the research supporting the no significant difference phenomenon [17].

In March 2020, Zimmerman [39] proposed that the transition from in-person to distance teaching offered a unique opportunity to examine how students “perform in these courses compared to the face-to-face kind, without worrying about self-selection bias.” The next day, George Veletsianos published on his webpage a reply to Zimmerman stating that this is a piece of bad advice for two reasons: the first was the large body of literature showing that there are “no significant differences between in-person and online courses.” The second said that online courses prepared in a week would not be as good as those prepared in months or even years.

Two weeks later, Thomas Tobin commented that if self-selection bias is a problem for students who follow online courses, it is also a problem for those who choose face-to-face programs: “The two types of instruction are apples and oranges” [40]. Finally, Hodges et al. [37] shifted the attention from methodology to terminology. Their article insisted that the type of instruction offered in response to the COVID-19 crisis should be named emergency remote teaching (ERT) because it has nothing in common with well-designed online courses. They also warned the educators that “The rapid approach necessary for emergency remote teaching may diminish the quality of the courses delivered.”

Hodges et al. drew a line between the experiences of the academic community since the pandemic outbreak and the idealisation of online learning. Synchronous remote teaching during the pandemic is different from asynchronous online learning. However, both of them are non-in-person learning modalities. According to Susan Ramlo, ERT impoverishes our understanding of the online experience [41] because it separates the latter from lessons learned during the pandemic. Besides, it does not clarify what research findings would serve as evidence for or against the claim regarding ERT quality. Students and teachers use the ERT experience to get an idea of their lives under online education. Research findings from different countries indicate a strong desire to return to classroom relationships. Are these findings irrelevant to the online proposal? Does the students’ verdict indicate that the rapidness of the transition from in-person to remote teaching diminished the quality of teaching? Research findings on students’ attitudes towards ERT offer valuable considerations regarding the prospects of online education in the post-COVID era.

## Past Research Findings on ERT

In July 2021, we searched the Scopus database using the keywords “emergency remote teaching” and “research.” The search returned 72 documents. Twenty-one of them were research articles recording students’ experiences and attitudes during ERT. Thirteen were based on quantitative research, seven on qualitative, and one on mixed methodology.

Quantitative studies published soon after the movement restrictions reported that students consider ERT useful for their studies [42, 43]. More recent publications have verified this conclusion [2, 44]. Petchamé et al. [44] compared students’ perceptions of Face-to-Face, ERT and Smart Classroom teaching during the pandemic and found that students perceived face-to-face classes as better than the other two options in most facets, except in the amount of time that students spend arriving at the University. Students reported better student–teacher interaction, a higher concentration level, and more effective teamwork when in the classroom. However, some academic teachers considered needing more resources and training [45] to perform effectively in the remote digital environment.

Recent publications have moved further from questions regarding the preference and effectiveness of learning during ERT to the nuances of students’ lives. Publications based on qualitative research [3, 27, 28, 41, 46] made more explicit some of the students’ experiences during ERT capturing their feelings during the pandemic. Despite the rigid procedures and centralisation characterising professional bureaucracies, university teachers responded flexibly to the new situation and moved away from conventional obligations to take care of the well-being of their students [47]. Research conducted at the University of San Diego found that students reported similar or lower stress levels and found their remote courses similarly or less challenging. The students spent approximately the same number of hours on their courses, and there were no notable increases in dropouts, failure rates, or disparities [47]. The faculty members were interested in the well-being of the students and made changes in their practices to ensure educational continuity [3]. Research has successfully captured the compassion and flexibility shown by faculty members to ease the students’ lives [27], while the students appreciated policy changes to classes and grading. Academic teachers took care of course continuity and the human aspect of teaching, responding promptly to questions, providing clear and transparent information, or connecting the students to resources or people to help them. “ERT created a physical divide between faculty and students, but it also required the faculty to pay more attention to their students’ personal lives” [27].

Several interesting issues emerged from the research conducted on ERT. Publications from different countries having different research objectives are summarised under the following headings: students’ preference, student–instructor interaction, student’s concentration/motivation, teamwork/interaction with peers, time to commute to the University, technical ease, workload, and emotions–loneliness–stress.

### Students’ Preference

A few publications communicated results showing preference or satisfaction with distance learning [47, 48]. In most of the studies, the students preferred in-person education. This was manifested as a lack of interest in continuing with the online mode [2]; positive emotions during face to face classes [44, 49]; preference for face to face albeit the initial enthusiasm with the online format [45]; significant reluctance towards emergency remote teaching from first-year students [50]; and problems with the withdrawal of the teacher in remote assessment [51].

### Student–Instructor Interaction

Students value direct communication with their teachers during face-to-face classes [44] regarding learning effectiveness and social exchanges between the community members. In that respect, the disruption of social relationships has affected both the students and the teachers [2]. Especially for the students, the unmediated social interactions (didactic and socio-relational) with instructors and peers [45] has been described as a reason for preferring the in-person modality. The students considered that the in-person courses enhance their ability to connect and interact with their teachers [46].

### Level of Concentration/Distraction, Motivation

Low motivation, low concentration, and distraction caused by the home environment are commonly reported problems during ERT. This was accompanied by difficulty to understand the content [2], low engagement with the course and the course material [46] and difficulty to remain engaged when studying at home [50, 52].

### Teamwork/Interaction with Peers

Face-to-face interaction makes teamwork more effective than virtual online teams. The students considered that non-face-to-face teaching does not benefit the communication between classmates [26], with the students in introductory classes being more affected [47]. Other problems were interference from family obligations, higher drop/fail rates in some classes [47], and the reduced ability of the students to interact with their colleagues [46].

### The Time Needed to Commute to the University

Whenever asked, the students express their pleasure for not commuting to the campus: students consider the amount of time needed to commute to the university negative [44]. The main three advantages of e-learning are time efficiency, convenience, and accessibility [52].

### Technical Ease

A practical recommendation that emerged during the pandemic was to record the lectures and make them available for future use: “recording and posting lectures and offering asynchronous or makeup exams and quizzes… could be carried over into non-pandemic quarters to help improve the student experience” [47]; “Recording class sessions in the Smart Classroom modality is considered a useful option” [44]; “the factor most highly ranked was watching recorded lecture videos” [46].

### Workload

Some articles commented on the increase in workload for both teachers and students [2]. Other publications reported lower stress and less time on the courses or an equal amount of work for the two modalities [47].

### Emotions–Loneliness–Stress

One of the most interesting findings in ERT literature is the students’ desire to restore their social relationships [50, 52]. This is expressed as a desire to return to the classrooms or frustration for losing contact with peers. This point has been raised mainly in qualitative research findings. Quantitative research based on models like the technology acceptance model usually misses this point’s details. The classroom environment offered a higher degree of positive feelings [44]. The switch to emergency remote teaching has been a stressful experience. The loss of interaction and communication with instructors and fellow students has been described as a source of frustration and diminished learning [46]. The students missed their academic rituals and interactions with peers and teachers. As Cernicova-Buca, and Dragomir notice, “students feel comfortable within human interaction (colleagues and not robots, familiar/class teacher, not other teachers)” [51]. The lack of human interaction and unmediated social relationships are the most significant factors affecting the psychological state of the students and the effectiveness of learning. The possibility of a total transformation of education through technology under the promise of more efficient learning includes risks of less learning not because of the lack of sophistication of the digital learning tools but because of the absence of human interaction and coherent social relationships. The consequences of such decisions require careful thinking since very little is known about their long-term effects [53].

To draw sound conclusions from the literature review, the various findings are understood within the context of the movement restrictions imposed and the concomitant feelings of stress, disappointment and frustration due to the disruption of the social relationships. In that respect, the non-preference for remote education is partly the result of frustration due to the disruption of normal social relationships during the pandemic. However, it conveys a message for the future, saying that separating learning from social experience undermines learning itself. The research findings of Petchamé et al. [44] are illuminating as they show that even the well-prepared and well-structured Smart Classroom did not attract the students’ preference compared to in-person teaching.

A second conclusion is the flexibility and adaptability of the academic teachers to the new situation despite the rigid procedures of the bureaucratic universities. The university teachers not only transferred their teaching material to digital form within a few days but also paid particular attention to supporting their students during the difficult period of movement restrictions. We speculate that the characteristics of the technological basis did not much affect teachers’ actions dictated by compassion and professionalism.

Despite the widely held beliefs that technology frees people from the excess workload, research findings have shown that the situation is the opposite. Digitisation of the material and the communication increased the workload for students and teachers.

The most important lesson learnt from the literature review is the positive influence of student–student and student–teacher face-to-face relationships in effective learning. However, teachers’ interest in students’ well-being cannot effectively combat feelings of isolation in the long run [47]. Current findings indicate that well-prepared distant teaching lags in terms of psychological support to the students [44] and the development of competencies like teamwork and peer-to-peer cooperation [2, 44, 46, 47]. Our interpretation of the literature review concludes that socialisation is not just a feature of education but a prerequisite for successful learning, and therefore learning outside the social context is a risky strategy [53].

Students consider the broader environment and their experiences during the global pandemic, and they miss classroom socialisation and learning [3]. Students do not perceive learning as distinct from the rest of their life. They adopt a holistic approach where learning is embedded in their social activities and is part of their social experiences. A life depleted from experiences of face-to-face communication is not the proper context for effective learning [54]—student engagement occurs when learning is integrated with meaningful social experiences.

## Methods

The data were collected using an anonymous questionnaire administered to the students via the Open eClass platform, an Integrated Course Management System offered by the Greek University Network (GUNET) to support asynchronous e-learning services. The respondents were full-time students of the Department of Electrical and Electronic Engineering, University of West Attica. A 25-item questionnaire was administered to gauge the students’ perceptions, attitudes, and experiences during ERT compared to face-to-face teaching. The questionnaire included one open-ended question, asking the respondents to express their views and feeling on the new learning reality. The data were collected between September and October 2020. The students were encouraged to fill out the questionnaire, but participation was voluntary. A total number of 336 students replied to the questionnaire.

Demographic data included gender, age, and year of enrolment. Thirty-nine per cent of the respondents were first-year students enrolled in 2020, 21% enrolled in 2019 (second year), 10% in 2018, 11% enrolled in 2017, and the rest 19% were students enrolled in 2016 or earlier. The first-year students did not have experience of in-person university lectures, but they had experienced remote teaching during the final year of their Lyceum studies. The gap between students’ expectations regarding university life and reality can cause anxiety [55], poor academic performance, and increased drop-out rates [56] if not managed successfully. The research method, the questionnaire, the theoretical underpinning of its items and the quantitative findings have been presented in another publication [6]. This publication focuses in the analysis of the text answers collected.

## Results and Discussion

The quantitative findings presented in a previous publication [6] show variations in the preference of the teaching modality with the year of studies. This article makes a brief reference to the quantitative results, focusing on the text answers of the respondents.

### The Questionnaire Findings

Overall, the majority (60%) preferred face-to-face teaching, 31% preferred remote teaching, and 9% expressed no particular preference. First and second-year students expressed a stronger preference for in-person teaching. Eighty-five percent of the first-year and 59% of the second-year students preferred face-to-face teaching, while 61% of the senior students (fourth or fifth year) preferred remote education.

The students were asked which modality they would choose if there were no restrictions. The percentages were close to those of preference: 63% would choose in-person, and 30% remote courses. However, 42% of the students replied that ERT was a pleasant solution given the movement restrictions and 33% characterized remote teaching as an unpleasant solution.

Overall, 77% of the respondents expressed positive feelings for not having to commute to the campus. This percentage was high (68%) even among the students who preferred in-person teaching, while it reached 93% among the respondents who preferred distance teaching. Not commuting to the campus was not a factor differentiating the students’ difference in modality preferences, and therefore it cannot explain the modality choices.

Table 1 shows the students’ replies on active participation during lectures, e.g. asking questions, expressing ideas easily, remaining concentrated to the lecture for longer, understanding, remaining concentrated during the classes, and more effective communication with the teacher.

**Table 1:** Class participation (*N* = 336)

	F2F (%)	Any (%)	RT (%)
Preference	60	9	31
Express ideas, ask questions more easily	44	34	22
More time concentrated	54	26	20
Understand better	55	29	16
More engaged with learning	53	32	15
More effective communication with tutor	51	32	17

Forty-four percent of the respondents reported that they express their ideas and ask questions more easily during in-person classes, while the respective percentage for remote teaching was 22%. The majority of the respondents replied that they remained more time concentrated, understand better, are more engaged with learning, and communicate more effectively with the teacher during in-person classes. Another 34% replied that the teaching modality does not influence class participation. These results varied with the year of studies. First-year students considered face-to-face teaching more effective in all aspects, followed by second-year ones. Overall the students considered that in-person teaching facilitates learning compared to remote classes.

The quantitative results showed that the students who prefer in-person teaching consider that this modality makes learning more effective. These students considered that during face-to-face classes, communication with the teacher is more effective (66%), understanding the material is better (81%), concentration is longer (73%), and better (76%), and expression of ideas is more straightforward (56%). The preference for face-to-face teaching was influenced by perceptions of effectiveness in learning and communication.

However, this is not the case with the students who prefer remote teaching. Only 33% of these students agreed that expressing ideas is easier during remote lectures. Only 27% considered that remoteness makes communication with the teacher more effective, 42% said they understand better, and 44% said they remain concentrated for longer. Overall their preference for remote teaching was not strongly influenced by perceptions of higher effectiveness of the particular modality in terms of learning, engagement, class participation, and communication.

Our findings show that a higher preference for remote teaching is not proof of superiority in terms of learning but is mainly related to pressures for accommodating life demands to education. Fitting the preferred teaching modality to the way of living was an essential factor of the two groups of students, 67% for those who preferred in-person teaching and 61% for those preferring remote. Compared with the percentages to the answers related to learning effectiveness, fitting education modality to life duties is more critical than learning effectiveness for students who prefer remote teaching.

### Text Answers

The text answers allowed students to explain their attitudes in their own words. The comments showed the perplexity of the modality preference question, particularly for the students who preferred remote teaching.

We received 82 text answers in total. Twenty-seven of them were from students with a preference for remote teaching, 50 from students with a preference for in-person teaching, and five from students who expressed no particular preference for any of the two modalities. Some of the students replied in a very emotional way. For example, two students commented on remote education: “It is horrible!”, (S121, Enrolment: 2020, preference: in-person). “At the beginning, it looked nice and welcomed, but soon it became too tiresome.” (S217, Enrolment: 2020, preference: in-person). A second-year student expressed a particularly negative view of this modality: “Every time I follow a remote lecture, I feel something is dying inside me. I have already failed in the past semester. I am afraid I will fail this semester despite my efforts to be consistent with my studies. Distance education is the worst thing that happened in my academic life.” (S218, Enrolment: 2019, preference: in-person). Opposite to these considerations, a third-year student noticed: “I dream of a day in the future when I will have the opportunity to complete a course even if I cannot, or I do not want to follow the lectures in-person” (S67, Enrolment: 2017, preference: remote).

The respondents commented on the following issues:

Socialisation (17 comments). The students expressed negative feelings for the lack of face-to-face communication during the classes, e.g., S12: “in-person teaching is superior because it allows direct interaction between the people.” (Enrolment: 2014, no-preference); S37: “Remote lectures don’t allow socialisation” (Enrolment: 2020, preference: remote); S67: “I miss socialising with my colleagues at the refectory, I miss the mushroom soup and tichou” (Enrolment 2017, preference: remote); S69: “Socialisation between the students is diminished” (Enrolment: 2016, preference: remote); S135: “Sadly, socialisation is reduced giving rise to addiction to technology” (Enrolment: 2020, preference: in-person); S143: “There are no social relationships and this is something we don’t like. The screen is not our friend and computer communication is not like face-to-face” (Enrolment: 2020, preference: in-person); S146: “I have the gut feeling, that this teaching modality (remote) will increase the distance between humans and accustom them to staying in front of a screen the whole day” (Enrolment: 2019, preference: in-person); S181: “The lack of direct contact with other persons during the day or during the lessons, generates feelings of isolation and alienation, affecting our psychological health” (Enrolment: 2017, preference: in-person).

Student–teacher communication (14 comments). Half of the comments made were from the students who preferred in-person learning. Quite interestingly, some students who preferred remote teaching commented on the importance of the student–teacher interaction, e.g., S51: “Remote learning is not like in-person, where one can communicate directly with the teacher.” (Enrolment: 2018, preference: remote); S169: “There is no face-to-face interaction with the teachers, and this makes teaching difficult for both the students and teachers” (Enrolment: 2018, preference: in-person); S81: “The absence of physical presence hampers the development of a relationship between the student and the teacher” (Enrolment: 2020, preference: remote).

Technical issues (13 comments), e.g., S100: “The biggest problem is the internet connection. It would be convenient if the lectures were recorded and uploaded on the LMS platform” (Enrolment: 2017, no preference).

Contingency issues (19 comments). e.g., S15: “Given the health risks because of the pandemic, remote lecturing is the best way to protect ourselves and the others” (Enrolment: 2019, preference: remote).

Fatigue/convenience (22 comments). Students commented on the convenience of not having to commute to the university, but also on the so-called “zoom fatigue” [57], e.g., S143: “(remote teaching) is convenient because we can do whatever we want behind the screen in the comfort and warmth of the home environment” (Enrolment: 2020, preference in-person); S109: “Remote teaching is very convenient for me because it allows me to work.” (Enrolment: 2019, preference: remote); S3: “Looking at the screen for long hours is not good for the eyes” (Enrolment:2020, no-preference); S72: “eye-fatigue is obvious after 5 or 6 hours of lectures” (Enrolment: 2016, preference: remote); S128 “At the end of an ordinary day I feel exhausted” (Enrolment: 2020, preference: in-person); S182: “being in front of a screen for 6 to 12 hours is bad for my physical and psychological health.” (Enrolment: 2017, preference: in-person).

Saving of money (4 comments). Some students linked “not having to commute” with saving money. e.g., S33: “I do not have to commute from the town I live to Athens. I do not spend money, and I do not get tired” (Enrolment: 2020, preference: remote).

Learning/understanding/concentration (7 comments). Most of these text answers considered the higher effectiveness of the classroom environment; e.g., S209: “The opportunity for deep quality discussions is limited because we spent a lot of time on technical issues related to the platform or questions on practical issues” (Enrolment: 2020, preference: in-person); S69: “It is easier to follow a remote lecture because there is no classroom noise and distance from the board” (Enrolment: 2016, preference: remote); S108: “In-person education is more effective because you have to follow a strict schedule of lectures and this is very helpful for people who have other responsibilities as well” (Enrolment: 2019, preference: in-person); S164: “There were too many students in the auditoria, and I lost contact with the lectures” (Enrolment: 2018, no preference); S114: “I want to follow the (remote) lectures, but I cannot get concentrated” (Enrolment 2020, preference: in-person); S160: “When students follow a lecture in the classroom, they remain concentrated because they know that their fellow students are trying as hard as they do” (Enrolment: 2019, preference: in-person);

Labs (7 comments). All the respondents agreed, irrespectively of the modality of preference, that remote labs are inefficient compared to on-site delivery; e.g., S89: “Doing the labs remotely is a disaster. During the previous semester, I followed the labs remotely, but I learned very few things.” (Enrolment: 2019, no preference); S108: “Doing labs remotely is ridiculous.” (Enrolment: 2019, in-person); S131: “Labs must be done with a physical presence.” (Enrolment: 2018, preference: remote).

Exams (14 comments). The students complained of not having enough time to complete their answers. The comments regarding distance exams were mixed. Some students questioned the fairness of the remote exams, while others happily commented on the possibility to pass the exams more easily; e.g., S108: “Distance examination is unfair, especially for the students who are consistent with their responsibilities. They cause much anxiety because they are impersonal.” (Enrolment: 2019, preference: in-person); S139: “Distance exams are less stressful because I can use my notes” (Enrolment: 2018, preference: in-person); S180: “Getting help from my friends is precious during the exams!” (Enrolment: 2019, preference: in-person); S157: “I have problems with the time given to answer the exam questions” (Enrolment: 2019, preference: in-person); S78: “… 80% of the exams are easier to pass (crucial to me)” (Enrolment: 2015, preference: remote).

### Discussion of the Text Answers

Analysis of the text answers illuminated aspects of students’ life during the period of the movement restrictions [30]. It provided a richer picture regarding the students’ preference towards the two modalities and raised the issues of remote examinations and labs.

Laboratory education is central in engineering studies, and it is a challenge to deliver remotely. Some publications attribute the observed lag of engineering education in adopting online teaching to the difficulties of remote lab education [58]. Students enrolled in 2019 or earlier raised the issue of remote lab education in their text answers. These students had the experience of on-site labs compared to the experience of remote synchronous labs. The respondents contrasted the two experiences and commented on the perceived ineffectiveness of the latter. Labs in electrical and electronic engineering involve psychomotor and sensory faculties, which are difficult to exercise remotely [59]. The students appreciated the teachers’ effort to ensure the continuation of lab education, but they considered that lack of direct contact with the apparatus undermines the essence of lab education.

Cheating during remote exams includes accessing resources, collusion, and impersonation. The transition from in-person to remote assessment added new problems to existing ones [60]. The students focused on inadequate time given during remote exams [61, 62], anxiety, and increased opportunities for cheating. For some students, the internet connection quality was a source of anxiety during the exams, while for others, the home environment and the teacher’s absence reduced stress and anxiety. Some of the respondents commented enthusiastically on the possibility of cheating during remote exams. Considerations regarding the easiness of remote exams appear to influence the modality preference [63].

Other publications have reported reduced stress when the examination is taken from home, accompanied by feelings of easiness for being alone without the teacher’s presence [51]. Recommendations for diminishing unfair practices [62] assume that remote exams are a manageable problem. However, as students’ replies showed, instructions for stricter time limits to prevent dishonest behaviours have generated more problems [64] than those intended to solve.

Understanding the students’ modality preference:

Student satisfaction was significantly lower during ERT because of the absence of in-person interaction with peers and teachers [65–67]. Even in periods of political unrest, when the design characteristics of online learning undermined students’ solidarity, teachers and students preferred working together, meeting at coffee shops, or elsewhere outside the university [68].

The students were concerned with the disruption of the social relationships when teaching moved online. Social relationships are influenced by the materiality of the environment [69]. The fast transition to remote teaching challenged the taken-for-granted materiality of in-person education, which included official learning activities (e.g., classrooms, labs), extracurricular activities (e.g., attending seminars, students’ union), and physical places (e.g., library, coffee shop) [70]. Students who had developed valuable relationships in the traditional university felt uneasy when obliged to confine themselves to the digital space of ERT. Some students expressed their appreciation for face-to-face interactions while preferring remote learning options [41].

Qualitative information collected via free-text answers provides student-specific details on the rationale of their choices. According to the text answers, preference for remote teaching is influenced by the need to accommodate the fulfilment of personal duties or needs with education. O’ Neil et al. [63] found that students with experience in online courses, students who avoid academic work and those competent in time management are more likely to take a course online. However, our quantitative findings indicate that preference for remote education is not strongly related to beliefs of more effective learning.

The quantitative and qualitative findings show that preference for remote teaching is triggered by pressures to accommodate personal needs, duties or personality characteristics with education, and it is less related to perceived effectiveness in learning. Students who already had a job were more positive towards remote education, hoping that, in the new environment, they would manage to continue their studies while working. One student who felt uncomfortable when surrounded by many people welcomed remote teaching (text answer 73).

Moreover, preference for remote teaching was related to the pressures or needs during a particular period of students’ lives. Belonging comprises four dimensions: affect, place, social relationships, and politics [70, 71]. Conceptualising belonging in a time–space context makes the transition from belonging to un-belonging more intelligible. Students may feel closer to the university space at a certain period of their life, attaching themselves to in-person teaching, while in another period, they may feel that the university is not an important setting for them to belong to [72, 73]. Some of the text answers emphasised the contextual factors (timing, family or job obligations) to explain their preference for remote teaching. Remote teaching is the preferable option in response to temporal and contextual pressures.

Balancing learning with personal responsibilities and duties was central for students who preferred remote teaching. Some of the students appreciated the importance of face-to-face interactions and explained their preference for remote teaching by referring to their obligations at the particular period of their lives. This is far from considering that the convenience of studying from home is attractive to all the students. The following text answers clarify this point: S12: “My preference for remote teaching is based on my duties during this period of my life. I prefer remote teaching because I save time for my job. However, in-person teaching is superior because it allows interaction between the people.” (Enrolment: 2014, no-preference); S51: “I have been in the classrooms for years… remote teaching is suitable for me now.” (Enrolment: 2018, preference: remote); S60: “The reason I prefer remote lectures during this period is that I have to pass some previous-semester exams. Remote education allows me to follow more classes.” (Enrolment: 2018, preference: remote); S73: “I get very anxious when people surround me, and remote lectures help me feel calm.” (Enrolment: 2016, preference: remote); S83: “Remote lectures are an opportunity for the students who have a job, and they would not come to the campus anyway, to follow the lectures.” (Enrolment: 2014, preference: remote).

The qualitative data collected show that students see ERT as an alternative modality and make complicated decisions based on the specific needs they face during a specific period of their student life. The importance of not commuting to the campus is challenged by the text answers collected [74, 75]. Although the vast majority of the respondents considered “not having to commute to the campus” attractive, only a tiny portion of them explained their preference on the basis of learning from the comfort of their home environment. Other students preferred ERT in response to practical difficulties they faced during a specific period of their life, such as family and job obligations or studies. Some of them preferred remote teaching to avoid in-person assessment [63]. Therefore, preference for remote teaching is not proof of the superiority of online education characteristics.

The effectiveness of learning is essential for the students who prefer in-person teaching. These students consider that class participation, concentration during the lectures and understanding of the taught material is more effective when classes are taken in person, and they explain their preference in terms of more effective learning. Their text answers indicate the importance they attribute to the cultivation of social relationships to increase student engagement and make learning and understanding more probable. However, the role of social relationships is not confined to effective learning. They also stressed the importance of socialisation for their psychological and mental health. Therefore, they consider learning and socialisation complementary aspects of education.

Preference for in-person teaching was primarily influenced by the belief that the classroom is a more effective learning environment [76]. Although not commuting to the university was considered attractive, the effectiveness of learning and socialisation were considered more important. These students value face-to-face relationships and consider their importance for learning and personality development. They also expressed worries regarding the prolonged absence of face-to-face communication and addiction to technology on psychological health and human relationships [77].

As described in the introduction, the objective of this publication is to understand the modality preference and the criteria of preference decisions. Our interpretation of the information collected indicates that the criteria underlying preference decisions are different for the two modalities. The survey results showed that the students who prefer in-person teaching consider this modality more effective in learning. However, only a minority of the students who prefer remote teaching consider that their preferred modality makes learning more effective.

Table 2 resumes the different preference criteria of the two groups of students. This list is not exhaustive, and further research can enrich our understanding of the differences between the two preferences. However, the findings of this publication indicate that students consider that the characteristics of classroom teaching facilitate learning, and for this reason, they prefer in-person teaching. For other students accommodating education with family and job obligations or health issues or convenience and comfort is more important, and consider remote teaching is the best option for them.

**Table 2:** Preference criteria

In-person	Remote
Effective earning	Fit education to personal duties or needs
Education as social experience	Education as utility
Collectivist	Individualistic

The students who prefer in-person teaching consider that the classroom environment, student–student and student–teacher direct communication, facilitates learning. They enjoy university socialisation which presumably satisfies their need of belonging. The situation is more complicated with the students who prefer remote education. Some of them appreciate the importance of in-person social relationships with their colleagues and teachers despite their preference for remote teaching. Others were detached from the university environment and preferred the comfort and convenience of the home environment. A third group admitted the importance of social relationships, but they explained that they did not need them during the current period of their life. Finally, some students explained their preferences based on the easier remote exams.

The students who preferred in-person teaching adopted a more collectivistic approach. They compared the two modalities on the basis of what is good or bad or what makes learning more effective for the students as a whole. The main focus of their answers was not on fulfilling particular individual needs. The majority of the in-person preference texts commented on the positive influence of socialisation and communication between teachers and students on learning. Two of the respondents adopted a more collegial view emphasising the role of a community of peers in learning. S160: “When students follow a lecture in the classroom, they remain concentrated because they know that their fellow students are trying as hard as they do” (Enrolment: 2019, preference: in-person); S199: “(during in-person classes) the students gather together and pursue a common goal. This generates a friendly environment.” (Enrolment: 2020, preference: in-person). These comments correspond to mutual focus attention, i.e., feelings of interpersonal solidarity generated when some people focus their attention on the same thing and are all aware of that [77].

The students who preferred remote teaching adopted a rather individualistic perspective in explaining their modality preference. Half of the text answers (14 out of 27) received from these students explained how remote teaching serves better some of their duties or needs. S19: “With remote teaching, … I have the comfort and the cleanliness of my home” (Enrolment:2017, preference: remote); S78: “I get up 5 min before the lecture. I have breakfast during the lesson. I can follow the lecture from any place in my home. I can make notes easily by taking screenshots. Finally, 80% of the exams are easier to pass (crucial to me).” (Enrolment:2015, preference: remote); S67: “Remote lectures are convenient. I do not have to commute to the campus and move from one classroom to another. My armchair is far more comfortable than the wooden seats of the classroom, where I get sweaty. The temperature at home is nice…” (Enrolment:2017, preference: remote); S37: “(Remote lectures) are convenient until I get my driving license.” (Enrolment:2020, preference: remote).

## Conclusion

Students who preferred in-person teaching focused on effective learning, and although they expressed their satisfaction for not commuting to the campus, they raised the problem of “zoom fatigue.” They were interested in preserving and developing face-to-face relationships with their colleagues and teachers. They stressed the importance of face-to-face interactions in learning, socialisation, and psychological health and viewed education in a collectivistic way rather than focusing on fulfilling their individual needs.

Students with a preference towards remote learning emphasised accommodating learning with other personal interests and duties and adopted a rather individualistic approach in their text answers. The students linked their preference for remote teaching to the fulfilment of duties facing at the particular period of their life, rather than an all-purpose any-time solution. In-person education was not considered less effective to remote while expressing worries about disrupting face-to-face relationships and “zoom fatigue.”

Views on maintaining social experiences and in-person interactions, and utility were identified among the respondents. ERT maintained order in Higher Education, although the students felt frustrated with the difficulty of carrying out satisfactory social relationships remotely.

## Data Availability

The datasets used and analyzed during the current study are available from the corresponding author on reasonable request.
